# Associations between Perceived Outdoor Environment and Walking Modifications in Community-Dwelling Older People: A Two-Year Follow-Up Study

**DOI:** 10.1177/0898264320944289

**Published:** 2020-07-28

**Authors:** Heidi Skantz, Taina Rantanen, Timo Rantalainen, Kirsi E. Keskinen, Lotta Palmberg, Erja Portegijs, Johanna Eronen, Merja Rantakokko

**Affiliations:** 1 4168 University of Jyväskylä, Jyväskylä, Finland; 2 4167 JAMK University of Applied Sciences, Jyväskylä, Finland

**Keywords:** aging, environment, compensation, mobility

## Abstract

**Objectives:** To examine associations of perceived outdoor environment with the prevalence and development of adaptive (e.g., slower pace) and maladaptive (e.g., avoiding walking) modifications in walking 2 km among older people. **Methods:** Community-dwelling 75–90 -year-old persons (*N* = 848) reported environmental outdoor mobility facilitators and barriers at baseline. Modifications in walking 2 km (adaptive, maladaptive, or no) were assessed at baseline and one and two years later. **Results:** Outdoor mobility facilitators were more often reported by those not using modifications or using adaptive versus maladaptive walking modifications. Differences in health and physical capacity explained most of the associations between outdoor mobility barriers and walking modifications. Perceived outdoor environment did not systematically predict future adaptive or maladaptive walking modifications. **Discussion:** Facilitators may compensate the declined physical capacity and alleviate the strain of walking longer distances by enabling the use of adaptive walking modifications, while lack of such facilitators fuels avoidance of walking longer distances.

In old age, declining functional ability increases vulnerability to environmental demands (Nahemow & Lawton, 1973). As environmental press increases, individuals may, decrease task demands and minimize losses in valued activities, modify their behavior or give up or reduce the frequency of doing a task (Freedman et al., 2016; Nahemow & Lawton, 1973; Skantz et al., 2019)*.* The first modifications are often seen in the most demanding physical tasks, such as walking longer distances (Mänty et al., 2007; Weiss et al., 2007).

Walking modifications are typical indicators of functional decline or preclinical disability (Fried et al., 2000). At the same time, some modifications may be adaptive and help individuals continue walking by reducing task demands, whereas other modifications may be maladaptive and lead to task avoidance (Skantz et al., 2019). We categorized self-reported modifications in walking 2 km distance into adaptive (e.g., reduced pace, using an aid, and resting in the middle) and maladaptive (reduced frequency or giving up doing the task). Adaptive walking modifications help to identify persons who strive to continue walking, whereas maladaptive walking modifications indicate avoidance, that is, having reduced or given up walking longer distances. In our previous study, the use of walking modifications that we termed adaptive postponed decline in life-space mobility and helped individuals maintain greater autonomy in outdoor participation, while the use of maladaptive walking modifications was associated with restrictions in outdoor mobility at baseline and over time (Skantz et al., 2019)*.* Selecting particular adaptation strategies may be conscious or subconscious (Lien et al., 2015) and may reflect, for example, a person’s capabilities, access to resources, preferred approach to perform an activity, and environmental opportunities (Baltes & Baltes, 1990; Gitlin et al., 2017; Tomey & Sowers, 2009). While previous studies have shown that person-related factors, such as older age and poorer functional ability, are associated with walking modifications (Freedman et al., 2016; Hoenig et al., 2006; Skantz et al., 2019), little attention has been paid to the associations between the outdoor environment and walking modifications. More specifically, it is not known how perceived facilitators for and barriers to environmental outdoor mobility relate to the use of adaptive and maladaptive walking modifications.

Based on the ecological model of aging (Nahemow & Lawton, 1973) and the disablement process model (Verbrugge & Jette, 1994), it can be hypothesized that perceptions of the outdoor environment are related to the use of adaptive and maladaptive walking modifications. These models indicate that as personal competencies decline with aging, walking performance can be maintained in three ways: reducing task demands, increasing the person’s capacity, or lowering environmental demands. In reality, assuming that their living environment affords opportunities for doing so, reducing task demands via adaptive walking modifications is most readily available strategy for people facing functional decline.

Specific environmental features can either support or hinder older people’s mobility. For example, depending on individuals’ functional capacity, hills in the nearby environment can facilitate walking for fitness for some and hinder walking for others (Eronen et al., 2014a; Sakari et al., 2017). Previous studies have shown that older people who perceive a higher number of environmental mobility facilitators, such as nature in the nearby environment or peaceful walkways, have higher physical activity levels and a lower risk for developing walking difficulty over time (Eronen et al., 2014a; Keskinen et al., 2018b; Portegijs et al., 2017a). Thus, we expect that for individuals facing functional decline, perceiving facilitators for outdoor mobility may increase their likelihood of using adaptive walking modifications and decrease their likelihood of using maladaptive walking modifications. In contrast, environmental demands that exceed a person’s capacity are risk factors for physical inactivity and the development of functional limitations over time (Keskinen et al., 2018a; Portegijs et al., 2017b; Rantakokko et al., 2011). Previous studies have shown that environmental barriers to outdoor mobility, such as poor street conditions or lack of resting places, are associated with restricted outdoor mobility (Rantakokko et al., 2015; Tsai et al., 2013) and increased the risk for developing walking difficulty over time (Keskinen et al., 2018a; Rantakokko et al., 2016). Thus, we expect that perceiving environmental barriers to outdoor mobility may especially be associated with the use of maladaptive walking modifications and increased risk for adopting maladaptive walking modifications over the follow-up among those not reporting such modifications at the baseline.

The aim of this study was to investigate whether perceived environmental outdoor mobility facilitators and barriers are associated with the use of adaptive and maladaptive walking modifications among community-dwelling older people. In addition, we investigated whether perceived environmental outdoor mobility facilitators and barriers predict the development of adaptive or maladaptive walking modifications over a 2-year follow-up.

## Methods

### Design and Study Participants

This study includes cross-sectional and longitudinal data drawn from the “Life-Space Mobility in Old Age” (LISPE) project, a 2-year prospective cohort study conducted between the years 2012 and 2014. The purpose of the LISPE study was to investigate the associations of the home and physical environment of older people with their health, functioning, disability, quality of life, and life-space mobility. A more detailed description of the LISPE study, including recruitment and nonrespondent analyses, has been reported previously (Rantanen et al., 2012)*.* Briefly, the study targeted community-dwelling people aged 75–90 years, randomly selected from the Finnish population register based on their age and residence in two municipalities: the city of Jyväskylä and the small town of Muurame (located in Central Finland). The study area is characterized by low hills, several lakes, rather quiet streets with predominantly residential traffic, and some busier streets with several intersections. The area contains several small parks with seating areas. Most of the shops and other services are concentrated in the municipal centers or subcenters. The residential areas comprise detached houses, row houses, and apartment buildings. Due to integrative planning and local housing policy, there is no clear socioeconomic differentiation between residential areas. Inclusion criteria were community-dwelling in the study area, willing to participate, and able to communicate and provide written informed consent. A total of 848 participants met the inclusion criteria and were interviewed face-to-face in their homes at baseline and followed up by telephone one (*n* = 816) and two (*n* = 761) years later. All interviews were conducted using structured computer-assisted personal interviewing. At the follow-ups, participants unable to answer questions via telephone were offered a face-to-face interview. The dropout rate over the 2-year follow-up period was 10%. The Ethical Committee of the University of Jyväskylä approved the LISPE study.

### Measurements

*Self-reported modifications in walking* 2* *km were assessed with a standardized questionnaire at baseline and at the 1- and 2-year follow-ups (Rantakokko et al., 2016). Walking modifications were investigated by asking participants whether they had modified their way of walking 2 km due to their health or physical functioning. Modifications were walking slower, resting during walking, using an aid, reducing frequency of walking, and having given up walking distances of 2 km. For each modification, participants were asked to state whether they used it (“yes” or “no”). In line with our previous categorization (Skantz et al., 2019), walking slower, resting during walking, and using an aid were categorized as adaptive modifications, as they indicate a striving to continue walking 2-km distances by reducing task demand. Those who reported adaptive walking modifications and reduced frequency of walking 2 km were also categorized as using adaptive walking modifications. Those who reported having given up walking 2 km or reducing their frequency of walking 2 km were, in the absence of adaptive modifications, categorized as using maladaptive modifications, as they indicate a reduced striving to continue the activity. Thus, we analyzed self-reported modifications in walking 2 km using the categories no modifications, adaptive modifications, and maladaptive modifications.

*Perceived environmental facilitators for outdoor mobility* were studied at baseline with a standardized questionnaire comprising 16 items selected based on our previous research (Rantakokko et al., 2015). Participants were asked to report all the items present in their living environment that they perceived as facilitating their outdoor mobility (present/absent). Environmental facilitators were categorized into three domains: *nature* (park or other green area, walking trail and skiing track, and nature and lakeside); *infrastructure* (good lighting, services close, even sidewalks, walkways without steep hills, resting places by the walking route, peaceful and good quality pedestrian routes, and safe crossings); and *safety* (appealing landscape, familiar surroundings, own yard, other people outdoors, no car traffic, and no cyclists on walkways) (Keskinen et al., 2019).

*Perceived environmental barriers to outdoor mobility* were also studied at baseline with a standardized questionnaire (Rantakokko et al., 2014) comprising 15 environmental barriers to outdoor mobility. Participants were asked to report all the features in their living environment that they perceived as hindering their outdoor mobility (present/absent). Environmental barriers were recoded into three domains: *nature* (hills in nearby environment and snow and ice in winter), *infrastructure* (poor street conditions, high curbs, lack of sidewalks, long distances to services, lack of benches during summer or winter, and poor lighting), and *safety* (noisy traffic, busy traffic, dangerous crossroads, vehicles on walkways, cyclists on walkways, and insecurity due to other pedestrians).

For the sensitivity analyses, participants were categorized based on their self-reported ability to independently walk 2 km (Mänty et al., 2007). Participants were considered unable to walk 2 km independently if they reported needing help or being unable to manage even with help.

#### Covariates

As covariates, we included variables that are associated with the use of walking modifications based on previous studies. Age and sex were obtained from national registers. Years of education, number of chronic conditions, depressive symptoms, lower extremity function, and ability to walk 2 km were assessed during the home interview. *Years of education,* as an indicator of socioeconomic status, was self-reported. The *Number of chronic conditions was* calculated from a list of 22 specified physician-diagnosed chronic conditions followed by an open-ended question on any other chronic diseases the participant might have (Portegijs et al., 2014). *Depressive symptoms* were assessed with the Center for Epidemiologic Studies Depression Scale (range 0–60; higher scores indicate more depressive symptoms) (Radloff, 1977). *Lower extremity function* was assessed with the short physical performance battery (SPPB) (Guralnik et al., 1994). For the sensitivity analyses, participants were categorized based on self-reported difficulties in walking 2 km (Mänty et al., 2007).

### Statistical Analyses

Baseline characteristics were described using means and standard deviations or percentages. Differences in the prevalence of perceived environmental outdoor mobility facilitators and barriers and in baseline characteristics between participants categorized according to their baseline walking modifications were tested with chi-square tests (χ^2^) and one-way analysis of variance. A Bonferroni test was used to compare means between participants using adaptive or maladaptive walking modifications. The sum of the environmental facilitators and barriers reported was calculated for each facilitator and barrier domain (nature, infrastructure, and safety) separately and then divided into those reporting 0, 1, and 2 or more facilitators or barriers. Analyses were run separately for each environmental facilitator and barrier domain (reporting 1 or ≥ 2 vs. 0) and for item-specific environmental facilitators for and barriers to outdoor mobility. The associations of perceived environmental outdoor mobility facilitators and barriers with walking modifications were assessed cross sectionally by using multinomial logistic regression analysis. The outcome variable was a nominal scale variable. Those with maladaptive walking modifications were used as a reference group when studying associations between environmental facilitators and categories of walking modifications. This was done to clarify whether the environmental facilitators reported by those using adaptive walking modifications differed from those using maladaptive walking modifications. In the analyses on environmental mobility barriers, those without walking modifications were used as a reference group. The cross-sectional models were first adjusted for age and sex and then, to control for individual differences, for age, sex, years of education, chronic conditions, depressive symptoms, and lower extremity function. Eight participants had missing information for years of education, four participants for depressive symptoms and nine participants for SPPB; these 21 participants were not included in the fully adjusted models.

In the longitudinal setting, logistic regression analyses were used to investigate the associations between perceived environmental outdoor mobility facilitators and barriers and the development of adaptive or maladaptive walking modifications. The development of adaptive walking modifications was studied among those who reported no walking modifications at baseline and who did not develop maladaptive modifications over the two-year follow-up period (*n* = 218). Participants who reported adaptive walking modifications at one or both follow-ups were defined having developed adaptive walking modifications. Similarly, the development of maladaptive walking modifications was studied only among those without maladaptive modifications at baseline (*n* = 610). Participants, who reported maladaptive walking modifications at one or both follow-ups, were defined as having developed maladaptive walking modifications. Analyses were conducted separately for each environmental subgroup (reporting 1 or ≥ 2 vs. no) and item-specific environmental facilitators for and barriers to outdoor mobility. All models were first adjusted for age and sex and then for age, sex, years of education, chronic conditions, depressive symptoms, and lower extremity function.

Finally, to test the robustness of our findings, we conducted further sensitivity analyses by excluding all participants unable to walk 2 km independently at baseline. This eliminated 112 participants from the maladaptive walking modifications category, four participants from the adaptive walking modifications category and one participant from the no walking modifications category. The sensitivity analyses were not performed for the development of adaptive walking modifications since all participants included in the model constructed from the whole sample were able to walk 2 km independently at baseline. False discovery rates (adjusted *p*-values) were calculated to correct for multiple testing to avoid type 1 error (Benjamini & Hochberg, 1995).

The results were regarded as statistically significant, if the 95% confidence intervals did not include one or the *p*-value was <.05. IBM SPSS version 24 for Windows (IBM Corp, Armonk, NY) and R version 3.6.1 (R Core Team, 2019) were used for statistical analyses.

## Results

### Participant Characteristics

The mean age of the study participants was 80.6 years (*N* = 848, age range 74.2–89.3, 62% women). At baseline, 38% (*n* = 325) used adaptive and 28% (*n* = 238) maladaptive modifications in walking 2 km. Those with no walking modifications (34%, *n* = 285) were younger, more often men, had more years of education and had fewer chronic conditions and depressive symptoms than those with adaptive or maladaptive walking modifications (*p* ≤ .011 for all variables; Table 1). Participants using adaptive walking modifications had intermediate scores in the health and physical capacity measurements compared to those with no walking modifications or with maladaptive walking modifications. Based on post hoc comparisons, statistically significant differences were observed between participants using adaptive and maladaptive walking modifications in all characteristics except for years of education (*p* = .170) and depressive symptoms (*p* = .056). For all participants, the most often reported facilitators for and barriers to outdoor mobility were nature related (Table 1). Of the individual items, nature in the nearby environment was the most reported facilitator for outdoor mobility (73%), whereas snow and ice in winter were the most often reported barriers to outdoor mobility (53%, Table 2). In general, those with maladaptive walking modifications reported fewer facilitators and more infrastructure barriers to outdoor mobility compared to those without walking modifications or with adaptive walking modifications. Participants with adaptive walking modifications reported more nature- or safety-related barriers to outdoor mobility than those using maladaptive walking modifications (Tables 1 and 2).

**Table 1.** Participant Characteristics and Proportion of Participants Reporting Outdoor Mobility Facilitators and Barriers in Subgroups by Modifications in Walking 2 km at Baseline (*N* = 848).

	No walking modifications (*n* = 285)	Adaptive walking modifications (*n* = 325)	Maladaptive walking modifications (*n* = 238)	*p*-value	Adjusted *p*-value
	Mean (*SD*)	Mean (*SD*)	Mean (*SD*)		
Age, years	78.9 (3.7)	80.9 (4.2)	82.3 (4.2)	**<.001** a	**<.001**
Age, range	74.2–89.1	74.2–89.3	74.4–89.2		
Education, years	10.3 (4.5)	9.5 (4.0)	8.8 (3.8)	**<.001** a	**<.001**
Chronic conditions, number	3.3 (2.0)	4.6 (2.4)	5.3 (2.5)	**<.001** a	**<.001**
SPPB, score	10.8 (1.4)	9.7 (2.0)	8.1 (3.3)	**<.001** a	**<.001**
CES-D, score	7.4 (5.8)	10.2 (6.3)	11.6 (7.9)	**<.001** a	**<.001**
	% (*n*)	% (*n*)	% (*n*)		
Women	54.0 (154)	64.3 (209)	68.5 (163)	**.002** b	**.011**
Unable to walk 2 km independently	.4 (1)	1.2 (4)	47.0 (112)	**<.001** b	**<.001**
Sum of nature facilitators				**<.001** b	**<.001**
0	8.1 (23)	16.9 (55)	22.3 (53)		
1	21.1 (60)	24.3 (79)	36.6 (87)		
≥2	70.9 (202)	58.8 (191)	41.2 (98)		
Sum of infrastructure facilitators				**.001** b	**.006**
0	21.8 (62)	21.2 (69)	33.3 (79)		
1	21.4 (61)	18.8 (61)	24.1 (57)		
≥2	56.8 (162)	60.0 (195)	42.6 (101)		
Sum of safety facilitators				**<.001** b	**<.001**
0	12.6 (36)	9.5 (31)	17.6 (42)		
1	10.9 (31)	20.3 (66)	20.6 (49)		
≥2	76.5 (218)	70.2 (228)	61.8 (147)		
Sum of nature barriers				**<.001** b	**<.001**
0	58.9 (168)	32.0 (104)	34.0 (81)		
1	33.3 (95)	44.0 (143)	42.9 (102)		
2	7.7 (22)	24.0 (78)	23.1 (55)		
Sum of infrastructure barriers				**<.001** b	**<.001**
0	74.4 (212)	52.6 (171)	45.0 (107)		
1	17.2 (49)	21.2 (69)	22.3 (53)		
≥2	8.4 (24)	26.2 (85)	32.8 (78)		
Sum of safety barriers				**.006** b	**.026**
0	75.8 (216)	64.3 (209)	75.6 (180)		
1	15.8 (45)	20.0 (65)	13.9 (33)		
≥2	8.4 (24)	15.7 (51)	10.5 (25)		

*Note.* SPPB = short physical performance battery; CES-D = Center for Epidemiologic Studies Depression Scale; *SD* = standard deviation. False discovery rates (adjusted *p*-values) were calculated to correct for multiple testing. Statistically significant values are bolded.

a Tested with one-way analysis of variance.

b Tested with chi-square test.

**Table 2.** Prevalence of Perceived Environmental Facilitators for and Barriers to Outdoor Mobility by Modifications in Walking 2 km at Baseline (*N* = 848).

	No walking modifications (*n* = 285)	Adaptive walking modifications (*n* = 325)	Maladaptive walking modifications (*n* = 238)	*p*-value	Adjusted *p*-value
Facilitators	% (*n*)	% (*n*)	% (*n*)		
Nature					
Park or other green area	43.2 (123)	42.5 (138)	34.9 (83)	.107	.244
Walking trail and skiing track	75.1 (214)	56.6 (184)	37.0 (88)	**<.001**	**<.001**
Nature and lakeside	80.7 (230)	71.7 (233)	64.3 (153)	**<.001**	**<.001**
Infrastructure					
Good lighting	43.9 (125)	40.6 (132)	25.6 (61)	**<.001**	**<.001**
Peaceful and good quality walkways	55.8 (159)	52.3 (170)	41.2 (98)	**.003**	**.014**
Even sidewalks	26.0 (74)	34.8 (113)	27.3 (65)	.038	.118
Resting places by the walking route	15.8 (45)	24.9 (81)	19.3 (46)	.018	.064
Walkways without steep hills	11.6 (33)	16.3 (53)	10.1 (24)	.065	.173
Services close	48.4 (138)	48.3 (157)	31.9 (76)	**<.001**	**<.001**
Safe crossings: traffic lights, zebra crossing, or traffic island between lanes	25.6 (73)	28.3 (92)	16.0 (38)	**.003**	**.014**
Safety					
Familiar environment	70.2 (200)	64.6 (210)	54.2 (129)	**.001**	**.006**
Appealing scenery	74.0 (211)	68.9 (224)	58.4 (139)	**.001**	**.006**
Own yard	55.8 (159)	58.2 (189)	58.0 (138)	.815	.893
Other people outdoors motivate	24.2 (69)	22.2 (72)	16.0 (38)	.060	.163
No car traffic	15.8 (45)	14.2 (46)	8.8 (21)	.052	.148
No cyclists on walkways	4.9 (14)	4.9 (16)	4.2 (10)	.907	.937
Barriers					
Nature					
Hills in the nearby environment	11.9 (34)	28.0 (91)	31.9 (76)	**<.001**	**<.001**
Snow and ice in winter	36.8 (105)	64.0 (208)	57.1 (136)	**<.001**	**<.001**
Infrastructure					
Poor street condition	15.1 (43)	21.5 (70)	19.3 (46)	.121	.257
High curbs	2.5 (7)	8.3 (27)	12.6 (30)	**<.001**	**<.001**
Lack of pedestrian zones	1.8 (5)	1.4 (12)	1.2 (10)	.227	.388
Long distances to services	4.2 (12)	9.2 (30)	24.4 (58)	**<.001**	**<.001**
Lack of resting places, summer	6.0 (17)	18.5 (60)	23.5 (56)	**<.001**	**<.001**
Lack of resting places, winter	7.4 (21)	24.0 (78)	25.2 (60)	**<.001**	**<.001**
Poor lighting	2.5 (7)	5.2 (17)	1.7 (4)	.041	.124
Safety					
Noisy traffic	1.8 (5)	5.5 (18)	3.8 (9)	.050	.145
Busy traffic	4.6 (13)	10.8 (35)	9.7 (23)	.015	.058
Dangerous crossroads	6.7 (19)	12.0 (39)	8.4 (20)	.066	.173
Vehicles on walkways	1.4 (4)	1.5 (5)	2.1 (5)	.807	.893
Cyclists in the walkways	16.8 (48)	23.7 (77)	14.7 (35)	.015	.058
Insecurity due to other pedestrians	4.6 (13)	7.1 (23)	4.2 (10)	.242	.406

*Note.* Tested with chi-square test. False discovery rates (adjusted *p*-values) were calculated to correct for multiple testing. Statistically significant values are bolded.

### Cross-Sectional Associations of Environmental Outdoor Mobility Facilitators with Walking Modifications

Older people reporting at least two nature- or infrastructure-related environmental facilitators had two to threefold higher odds for using no walking modifications compared to those using maladaptive walking modifications (adjusted for age, sex, years of education, chronic conditions, depressive symptoms, and lower extremity function; Table 3). Similarly, at least two infrastructure (OR 2.4, 95% CI 1.6–3.7) or safety-related (OR 2.5, 95% CI 1.4–4.3) facilitators for outdoor mobility were more likely to be reported by those using adaptive walking modifications than those using maladaptive walking modifications. In the item-specific analyses, participants who perceived a walking trail or a skiing track as a facilitator for outdoor mobility had almost fourfold higher odds for reporting no walking modifications than those reporting maladaptive walking modifications. Most of the infrastructure-related facilitators, such as good lighting or walkways without steep hills, were more commonly associated with those using adaptive than maladaptive walking modifications even when adjusted for all the covariates. Perceiving a walking trail or a skiing track (nature-related facilitators) and a familiar environment (safety-related facilitator) as facilitators was also associated with those using adaptive rather than maladaptive walking modifications.

**Table 3.** Cross-Sectional Associations of Perceived Environmental Facilitators for Outdoor Mobility with Walking Modifications in Community-Dwelling Older People. Odds are Reported for those with No Modifications (*n* = 281) and Adaptive Modifications (*n* = 319) versus Maladaptive Modifications (*n* = 227, reference).

Facilitator	Model 1				Model 2			
	No walking modifications (*n* = 281)		Adaptive walking modifications (*n* = 319)		No walking modifications (*n* = 281)		Adaptive walking modifications (*n* = 319)	
	OR (95% CI)	Adjusted *p*-value	OR (95% CI)	Adjusted *p*-value	OR (95% CI)	Adjusted *p*-value	OR (95% CI)	Adjusted *p*-value
Sum of nature facilitators								
1 versus 0	1.4 (.8–2.6)	.434	.8 (.5–1.4)	.636	1.1 (.5–2.2)	.893	1.5 (.9–2.4)	.356
≥2 versus 0	**3.8 (2.1–6.7)**	**<.001**	1.7 (1.1–2.7)	.056	**2.9 (1.5–5.6)**	**.006**	.7 (.4–1.2)	.256
Sum of infrastructure facilitators								
1 versus 0	1.5 (.9–2.6)	.221	1.3 (.8–2.1)	.479	1.8 (1.0–3.2)	.179	1.5 (.9–2.5)	.305
≥2 versus 0	**2.3 (1.5–3.5)**	**<.001**	**2.3 (1.5–3.5)**	**<.001**	**2.5 (1.5–4.2)**	**<.001**	**2.4 (1.6–3.7)**	**<.001**
Sum of safety facilitators								
1 versus 0	.7 (.4–1.4)	.466	1.8 (1.0–3.3)	.113	.7 (.4–1.6)	.630	2.0 (1.0–3.7)	.119
≥2 versus 0	1.8 (1.1–3.0)	.078	**2.1 (1.3–3.6)**	**.014**	1.9 (1.1–3.6)	.094	**2.5 (1.4–4.3)**	**.006**
Item specific								
Nature								
Park or other green area	1.4 (1.0–2.1)	.131	1.4 (1.0–2.0)	.141	1.4 (.9–2.1)	.285	1.4 (.9–2.0)	.244
Walking trail and skiing track	**4.5 (3.0–6.6)**	**<.001**	**2.1 (1.5–3.0)**	**<.001**	**3.7 (2.4–5.8)**	**<.001**	**1.9 (1.3–2.8)**	**.006**
Nature and lakeside	**1.9 (1.3–2.9)**	**.011**	1.3 (.9–1.9)	.264	1.6 (.9–2.6)	.162	1.2 (.8–1.7)	.581
Infrastructure								
Good lighting	**2.1 (1.4–3.0)**	**<.001**	**1.9 (1.3–2.7)**	**.004**	**1.9 (1.2–2.8)**	**.041**	**1.8 (1.2–2.6)**	**.018**
Peaceful and good quality walkways	**1.9 (1.3–2.7)**	**.004**	**1.6 (1.1–2.2)**	**.023**	**2.0 (1.3–3.0)**	**.011**	**1.8 (1.2–2.6)**	**.011**
Even sidewalks	.9 (.6–1.4)	.896	1.4 (1.0–2.1)	.136	1.2 (.7–1.8)	.718	1.6 (1.1–2.4)	.063
Resting places by the walking route	1.0 (.6–1.5)	.922	1.5 (1.0–2.3)	.132	1.3 (.7–2.2)	.582	1.6 (1.1–2.6)	.096
Walkways without steep hills	1.4 (.8–2.4)	.447	1.9 (1.1–3.1)	.055	2.1 (1.0–4.2)	.118	**2.4 (1.4–4.3)**	**.011**
Services close	**2.2 (1.5–3.2)**	**<.001**	**2.0 (1.4–2.9)**	**<.001**	**2.0 (1.3–3.1)**	**.011**	**1.9 (1.3–2.7)**	**.006**
Safe crossings: traffic lights, zebra crossing, or traffic island between lanes	1.7 (1.1–2.7)	.058	**2.0 (1.3–3.1)**	**.004**	1.5 (.9–2.6)	.244	**1.9 (1.2–3.0)**	**.022**
Safety								
Familiar environment	**2.3 (1.5–3.3)**	**<.001**	**1.6 (1.1–2.3)**	**.020**	2.3 (1.5–3.6)	.162	**1.7 (1.2–2.5)**	**.018**
Appealing scenery	**2.0 (1.3–2.9)**	**.004**	1.6 (1.1–2.2)	.045	**2.0 (1.3–3.2)**	**.014**	1.6 (1.1–2.3)	.067
Own yard	.9 (.6–1.3)	.722	1.0 (.7–1.4)	.992	.8 (.5–1.2)	.461	1.1 (.7–1.5)	.872
Other people outdoors motivate	1.6 (1.0–2.5)	.112	1.5 (.9–2.3)	.174	1.9 (1.1–3.3)	.060	1.6 (1.0–2.6)	.138
No car traffic	1.7 (.9–3.1)	.134	1.6 (.9–2.8)	.178	1.8 (.9–3.4)	.192	1.7 (.9–3.0)	.184
No cyclists on walkways	1.1 (.4–2.6)	.926	1.2 (.5–2.6)	.853	1.1 (.4–2.9)	.937	1.2 (.5–2.7)	.872

*Note.* Multinomial logistic regression analyses. Reference category: maladaptive walking modifications, *n* = 227. Model 1: Adjusted for age and sex. Model 2: Adjusted for age, sex, years of education, depressive symptoms, chronic conditions, and lower extremity function. OR = odds ratio; CI = confidence interval. False discovery rates (adjusted *p*-values) were calculated to correct for multiple testing. Statistically significant values are bolded.

### Cross-Sectional Associations of Environmental Outdoor Mobility Barriers with Walking Modifications

Participants reporting at least two infrastructure-related environmental barriers had increased odds for using adaptive (OR 2.5, 95% CI 1.4–4.2) or maladaptive (OR 2.3, 95% CI 1.3–4.2) walking modifications compared to those reporting no walking modifications (Table 4). Reporting one or two nature-related environmental barriers increased the odds for using adaptive but not maladaptive walking modifications when compared to those using no walking modifications. Of the individual mobility barriers, reporting hills in the nearby environment (OR 2.0, 95% CI 1.2–3.2), snow and ice during winter (OR 2.2, 95% CI 1.6–3.2) or lack of resting places in winter (OR 2.3, 95% CI 1.3–4.0) were more common among people using adaptive walking modifications than among those using no walking modifications. In contrast, reporting long distances to services (OR 4.5, 95% CI 2.1–9.6) was related to the use of maladaptive walking modifications. Safety-related barriers to outdoor mobility were not associated with the use of adaptive or maladaptive walking modifications when the models were adjusted for all the covariates.

**Table 4.** Cross-Sectional Associations of Perceived Environmental Barriers to Outdoor Mobility with Walking Modifications in Community-Dwelling Older People. Odds are Reported for those with Adaptive Modifications (*n* = 319) and Maladaptive Modifications (*n* = 227) versus those with No Modifications (*n* = 281, reference).

Barrier	Model 1				Model 2			
	Adaptive walking modifications (*n* = 319)		Maladaptive walking modifications (*n* = 227)		Adaptive walking modifications (*n* = 319)		Maladaptive walking modifications (*n* = 227)	
	OR (95% CI)	Adjusted *p*-value	OR (95% CI)	Adjusted *p*-value	OR (95% CI)	Adjusted *p*-value	OR (95% CI)	Adjusted *p*-value
Sum of nature barriers								
1 versus 0	**2.3 (1.6–3.3)**	**<.001**	**2.1 (1.4–3.1)**	**<.001**	**1.8 (1.2–2.7)**	**.014**	1.2 (.8–1.9)	.609
2 versus 0	**5.0 (1.6–3.3)**	**<.001**	**4.2 (2.3–7.5)**	**<.001**	**3.5 (2.0–6.2)**	**<.001**	2.0 (1.0–3.8)	.128
Sum of infrastructure barriers								
1 versus 0	1.6 (1.1–2.5)	.065	**1.9 (1.2–3.1)**	**.023**	1.4 (.9–2.1)	.339	1.3 (.8–2.3)	.494
≥2 versus 0	**3.8 (2.3–6.3)**	**<.001**	**5.1 (3.0–8.6)**	**<.001**	**2.5 (1.4–4.2)**	**.006**	**2.3 (1.3–4.2)**	**.029**
Sum of safety barriers								
1 versus 0	1.5 (1.0–2.3)	.155	.9 (.5–1.5)	.775	1.3 (.8–2.0)	.500	.7 (.4–1.3)	.468
≥2 versus 0	**2.1 (1.3–3.7)**	**.018**	1.2 (.6–2.2)	.730	1.4 (.8–2.4)	.494	.6 (.3–1.2)	.305
Item specific								
Nature								
Hills in the nearby environment	**2.6 (1.7–4.0)**	**<.001**	**3.0 (1.8–4.7)**	**<.001**	**2.0 (1.2–3.2)**	**.018**	1.9 (1.1–3.3)	.060
Snow and ice in winter	**2.8 (2.0–4.0)**	**<.001**	**2.0 (1.4–3.0)**	**<.001**	**2.2 (1.6–3.2)**	**<.001**	1.2 (.8–1.8)	.644
Infrastructure								
Poor street condition	1.4 (.9–2.2)	.194	1.2 (.7–1.9)	.626	1.1 (.7–1.7)	.911	.7 (.4–1.2)	.291
High curbs	**3.1 (1.3–7.4)**	**.028**	**4.6 (1.9–11.0)**	**.004**	1.7 (.6–4.4)	.472	1.2 (.4–3.3)	.872
Lack of pedestrian zones	2.2 (.8–6.6)	.242	2.7 (.9–8.5)	.159	2.9 (.9–9.5)	.210	3.6 (1.0–13.3)	.162
Long distances to services	2.0 (1.0–4.1)	.111	**6.1 (3.1–11.9)**	**<.001**	1.8 (.8–3.8)	.275	**4.5 (2.1–9.6)**	**<.001**
Lack of resting places, summer	**3.1 (1.7–5.5)**	**<.001**	**3.9 (2.2–7.1)**	**<.001**	2.0 (1.1–3.7)	.085	2.1 (1.1–4.0)	.094
Lack of resting places, winter	**3.4 (2.0–5.7)**	**<.001**	**3.2 (1.8–5.6)**	**<.001**	**2.3 (1.3–4.0)**	**.018**	1.8 (1.0–3.3)	.178
Poor lighting	2.3 (.9–5.8)	.151	.7 (.2–2.6)	.755	1.7 (.6–4.7)	.447	.4 (.1–1.7)	.380
Safety								
Noisy traffic	3.2 (1.2–8.9)	.065	2.2 (.7–6.9)	.282	2.1 (.7–6.3)	.348	1.5 (.4–5.3)	.730
Busy traffic	**2.5 (1.3–4.8)**	**.028**	**2.2 (1.1–4.6)**	.094	1.8 (.9–3.8)	.229	1.5 (.7–3.4)	.502
Dangerous crossroads	1.8 (1.0–3.3)	.106	1.2 (.6–2.4)	.722	1.4 (.8–2.6)	.473	.9 (.4–1.8)	.840
Vehicles on walkways	1.1 (.3–4.4)	.922	1.5 (.4–6.3)	.726	.8 (.2–3.4)	.872	.7 (.1–3.9)	.864
Cyclists in the walkways	1.4 (.9–2.2)	.182	.8 (.5–1.3)	.428	1.1 (.7–1.8)	.809	.5 (.3–.9)	.064
Insecurity due to other pedestrians	1.6 (.8–3.3)	.288	1.0 (.4–2.4)	.992	1.0 (.5–2.2)	.981	.4 (.2–1.2)	.257

*Note.* Multinomial logistic regression analyses. Reference category: no walking modifications. Model 1: Adjusted for age and sex. Model 2: Adjusted for age, sex, years of education, depressive symptoms, chronic conditions, and lower extremity function. OR = odds ratio; CI = confidence interval. False discovery rates (adjusted *p*-values) were calculated to correct for multiple testing. Statistically significant values are bolded.

Finally, to test the robustness of our findings, we conducted sensitivity analyses by excluding participants who reported being unable to walk 2 km independently at baseline. The results showed that while most of the associations between environmental facilitators and walking modifications disappeared (Supplementary Table 1), no changes were observed in the associations between environmental barriers and walking modifications (Supplementary Table 2).

### Longitudinal Associations of Environmental Outdoor Mobility Facilitators and Barriers with Walking Modifications

Of the 218 participants without walking modifications at baseline, 51.4% (*n* = 112) developed adaptive walking modifications during the 2-year follow-up period. No associations between environmental outdoor mobility facilitators and the development of adaptive walking modifications over time were observed (Table 5). Perceiving more than two infrastructure-related barriers and perceiving lack of resting places as a barrier to outdoor mobility increased the odds for using adaptive walking modifications over the follow-up in the age- and sex-adjusted model but not in the fully adjusted model (Table 6).

**Table 5.** Perceived Environmental Facilitators for Outdoor Mobility as Predictors of Use of Adaptive or Maladaptive Walking Modifications over 2-Year Follow-Up in Community-Dwelling Older People.

Facilitator	Adaptive walking modifications (*N* = 218)^a^				Maladaptive walking modifications (*N* = 610)^b^			
	Model 1		Model 2		Model 1		Model 2	
	OR (95% CI)	Adjusted *p*-value	OR (95% CI)	Adjusted *p*-value	OR (95% CI)	Adjusted *p*-value	OR (95% CI)	Adjusted *p*-value
Sum of nature facilitators								
1 versus 0	.8 (.3–2.5)	.810	.4 (.1–1.2)	.906	.7 (.4–1.2)	.282	.7 (.4–1.2)	.305
≥2 versus 0	.4 (.1–1.4)	.248	.7 (.3–1.8)	.339	1.0 (.5–1.7)	.926	1.0 (.5–1.8)	.977
Sum of infrastructure facilitators								
1 versus 0	.9 (.4–1.8)	.810	.7 (.4–1.3)	.406	.9 (.6–1.4)	.863	.8 (.5–1.3)	.893
≥2 versus 0	1.0 (.4–2.3)	.977	.6 (.3–1.3)	.819	.9 (.5–1.5)	.791	.9 (.5–1.6)	.937
Sum of safety facilitators								
1 versus 0	2.0 (.8–5.5)	.264	1.5 (.6–3.3)	.305	1.0 (.6–1.8)	.992	.9 (.5–1.6)	.872
≥2 versus 0	2.8 (.8–10.0)	.198	1.2 (.4–3.6)	.217	1.9 (1.0–3.8)	.128	1.7 (.8–3.4)	.288
Item specific								
Nature								
Park or other green area	1.2 (.7–2.2)	.650	1.0 (.5–2.0)	.977	.8 (.6–1.1)	.268	.8 (.5–1.1)	.872
Walking trail and skiing track	.8 (.4–1.7)	.735	.9 (.4–1.8)	.840	**.5 (.4–.7)**	**<.001**	**.5 (.3–.7)**	**<.001**
Nature and lakeside	1.8 (.8–3.8)	.264	2.0 (.8–4.8)	.256	1.3 (.8–1.9)	.428	1.3 (.8–2.0)	.406
Infrastructure								
Good lighting	.9 (.5–1.5)	.730	.8 (.4–1.5)	.582	.9 (.6–1.3)	.730	.9 (.6–1.3)	.671
Peaceful and good quality walkways	.9 (.5–1.7)	.911	.9 (.5–1.7)	.818	.9 (.6–1.2)	.620	.8 (.6–1.2)	.502
Even sidewalks	.9 (.5–1.8)	.896	.7 (.3–1.5)	.553	1.3 (.9–1.8)	.354	1.2 (.8–1.7)	.579
Resting places by the walking route	2.1 (.9–4.5)	.152	1.1 (.5–2.7)	.893	1.5 (1.0–2.3)	.136	1.3 (.9–2.0)	.386
Walkways without steep hills	1.9 (.8–4.8)	.270	1.9 (.7–5.4)	.380	1.2 (.7–1.9)	.650	1.1 (.7–1.9)	.809
Services close	1.0 (.6–1.8)	.992	.9 (.5–1.7)	.809	1.2 (.8–1.7)	.525	1.2 (.8–1.7)	.502
Safe crossings: traffic lights, zebra crossing, or traffic island between lanes	.9 (.5–1.8)	.903	.6 (.3–1.2)	.288	.9 (.6–1.3)	.623	.8 (.5–1.2)	.502
Safety								
Familiar environment	1.4 (.7–2.6)	.472	1.3 (.7–2.6)	.643	1.0 (.7–1.4)	.992	1.0 (.7–1.5)	.977
Appealing scenery	1.4 (.7–2.8)	.539	1.5 (.7–3.4)	.502	.8 (.5–1.1)	.245	.8 (.5–1.1)	.305
Own yard	1.1 (.6–2.0)	.883	1.2 (.6–2.3)	.809	1.1 (.8–1.6)	.737	1.1 (.8–1.6)	.796
Other people outdoors motivate	1.4 (.7–2.7)	.479	.8 (.4–1.8)	.809	1.0 (.6–1.4)	.910	.8 (.5–1.3)	.555
No car traffic	1.3 (.6–2.8)	.695	1.0 (.4–2.4)	.977	1.0 (.6–1.6)	.730	.9 (.5–1.4)	.787
No cyclists on walkways	2.7 (.7–9.7)	.237	2.3 (.6–9.4)	.409	1.2 (.5–2.6)	.815	1.1 (.5–2.4)	.937

*Note.* Development of adaptive and maladaptive walking modifications was analyzed in separate models by using binary logistic regression models. Model 1: Adjusted for age and sex. Model 2: Adjusted for age, sex, years of education, depressive symptoms, chronic conditions, and lower extremity function. OR = odds ratio; CI = confidence interval. False discovery rates (adjusted *p*-values) were calculated to correct for multiple testing. Statistically significant values are bolded.

a Reference category: no walking modifications.

b Reference category: no and adaptive walking modifications.

**Table 6.** Perceived Environmental Barriers to Outdoor Mobility as Predictors of Use of Adaptive or Maladaptive Walking Modifications over 2-Year Follow-Up in Community-Dwelling Older People.

Barrier	Adaptive walking modifications (*N* = 218)^a^				Maladaptive walking modifications (*N* = 610)^b^			
	Model 1		Model 2		Model 1		Model 2	
	OR (95% CI)	Adjusted *p*-value	OR (95% CI)	Adjusted *p*-value	OR (95% CI)	Adjusted *p*-value	OR (95% CI)	Adjusted *p*-value
Sum of nature barriers								
1 versus 0	1.0 (.3–3.2)	.992	.3 (.1–1.3)	.244	**2.4 (1.4–3.9)**	**.004**	1.9 (1.1–3.2)	.058
2 versus 0	1.2 (.7–2.3)	.726	1.0 (.5–2.0)	.937	**1.7 (1.2–2.5)**	**.023**	1.4 (.9–2.1)	.244
Sum of infrastructure barriers								
1 versus 0	**3.1 (1.0–9.7)**	.114	1.2 (.3–4.4)	.872	1.6 (1.0–2.5)	.116	1.3 (.8–2.1)	.502
≥2 versus 0	.8 (.4–1.8)	.770	.5 (.2–1.2)	.244	1.2 (.7–1.8)	.722	1.0 (.6–1.6)	.971
Sum of safety barriers								
1 versus 0	1.9 (.7–5.1)	.298	.7 (.3–1.8)	.923	1.2 (.7–2.0)	.728	.9 (.5–1.6)	.872
≥2 versus 0	.6 (.3–1.4)	.372	.3 (.1–.8)	.076	1.4 (.9–2.3)	.198	1.3 (.8–2.1)	.384
Item specific								
Nature								
Hills in the nearby environment	1.6 (.7–4.1)	.446	.9 (.3–2.4)	.872	1.7 (1.1–2.5)	.056	1.5 (1.0–2.3)	.185
Snow and ice in winter	1.0 (.5–1.7)	.926	.6 (.3–1.3)	.339	**1.8 (1.3–2.6)**	**.004**	1.5 (1.1–2.2)	.093
Infrastructure								
Poor street condition	.9 (.4–2.0)	.883	.5 (.2–1.4)	.333	1.5 (1.0–2.3)	.136	1.3 (.8–2.0)	.406
High curbs	6.0 (.6–63.5)	.240	6.3 (.4–98.6)	.340	1.7 (.9–3.5)	.237	1.2 (.6–2.6)	.809
Lack of pedestrian zones	1.8 (.3–11.6)	.713	1.2 (.1–12.1)	.923	1.0 (.3–3.1)	.992	1.3 (.4–4.2)	.809
Long distances to services	1.1 (.3–4.0)	.977	.3 (.1–1.5)	.305	1.0 (.5–1.9)	.992	1.0 (.5–1.9)	.946
Lack of resting places, summer	**7.3 (1.5–35.3)**	**.040**	3.7 (.7–19.1)	.261	1.4 (.8–2.3)	.354	1.1 (.6–1.9)	.872
Lack of resting places, winter	2.6 (.7–9.2)	.248	1.2 (.3–4.7)	.893	1.5 (1.0–2.4)	.160	1.2 (.7–2.0)	.607
Poor lighting	1.3 (.2–8.0)	.896	.7 (.1–6.7)	.872	1.2 (.5–2.9)	.755	.9 (.4–2.2)	.872
Safety								
Noisy traffic	1.4 (.2–10.5)	.854	.6 (.1–5.6)	.809	1.0 (.4–2.5)	.992	.7 (.3–1.9)	.667
Busy traffic	1.0 (.2–4.0)	.992	.8 (.2–3.8)	.872	2.0 (1.1–3.6)	.082	1.7 (.9–3.2)	.256
Dangerous crossroads	3.7 (1.0–12.7)	.107	2.7 (.7–10.8)	.321	1.3 (.7–2.2)	.610	1.1 (.6–1.9)	.893
Vehicles on walkways	.5 (.1–5.8)	.727	.2 (.0–3.5)	.450	.9 (.2–3.5)	.910	.8 (.2–3.4)	.872
Cyclists in the walkways	.8 (.4–1.7)	.730	.4 (.2–1.1)	.173	1.3 (.8–1.9)	.446	1.2 (.7–1.8)	.704
Insecurity due to other pedestrians	2.5 (.8–8.0)	.237	.9 (.2–3.3)	.893	1.0 (.5–2.1)	.992	.8 (.4–1.7)	.809

*Note.* Development of adaptive and maladaptive walking modifications was analyzed in separate models by using binary logistic regression models. Model 1: adjusted for age and sex. Model 2: adjusted for age, sex, years of education, depressive symptoms, chronic conditions, and lower extremity function. OR = odds ratio; CI = confidence interval. False discovery rates (adjusted *p*-values) were calculated to correct for multiple testing. Statistically significant values are bolded.

a Reference category: no walking modifications.

b Reference category: no and adaptive walking modifications.

Of the 610 participants who did not report maladaptive walking modifications at baseline, 22.3% (*n* = 136) developed maladaptive walking modifications during the 2-year follow-up period. Perceiving a walking trail or skiing track as a facilitator for outdoor mobility protected against the adoption of maladaptive walking modifications even when adjusted for age, sex, years of education, chronic conditions, depressive symptoms, and lower extremity function (OR .5, 95% CI .3–.7, Table 5). Otherwise, no associations were observed between perceived environmental facilitators and the development of maladaptive walking modifications. Reporting snow and ice in winter (OR 1.8, 95% CI 1.3–2.6) as barriers to outdoor mobility at baseline increased the odds for developing maladaptive walking modifications over time in the age- and sex-adjusted model (Table 6). However, the associations disappeared when all the covariates were added in the models. In the prospective sensitivity analyses, the exclusion of participants unable to walk 2 km independently at baseline did not change the longitudinal results (Supplementary Tables 3 and 4).

## Discussion

The present findings suggest that perceived environmental features coincide with, rather than consistently preceding, walking modifications. Perceiving environmental facilitators for outdoor mobility was associated with the use of no walking modifications or adaptive walking modifications rather than with the use of maladaptive walking modifications, whereas perceiving environmental barriers to outdoor mobility increased the odds for using both adaptive and maladaptive walking modifications in the age- and sex-adjusted models. There are several plausible reasons for the different associations found between perceived environmental outdoor mobility facilitators and adaptive and maladaptive walking modifications. Perceiving environmental outdoor mobility facilitators may serve as a motivation or enabler for individuals to adopt strategies that allow them to continue rather than reduce or give up walking longer distances, even when experiencing functional decline (Portegijs et al., 2017a). For example, infrastructural mobility facilitators may compensate for the decline in physical capacity and alleviate the strain of walking longer distances by enabling the use of adaptive walking modifications, while the lack of such facilitators may fuel lower frequency of or giving up walking longer distances, that is, maladaptive walking modifications stemming from the absence of perceived opportunities to reduce the task demands of walking longer distances. The use of maladaptive walking modifications may indicate that the task demands exceed personal capacity, potentially leading to reduced striving to continue the activity (Nahemow & Lawton, 1973). Thus, long distances to services can be considered an excessively demanding task demand for older people with poor physical capacity.

The current findings accord with those of previous studies showing that perceiving environmental facilitators is associated with higher physical activity levels (Barnett et al., 2017; Cerin et al., 2017; Keskinen et al., 2019). Further support for environmental mobility facilitators as motivators of outdoor mobility was provided by the present multinomial logistic regression analysis. In the model, those who reported environmental facilitators for outdoor mobility had higher odds for using no or adaptive walking modifications than those using maladaptive walking modifications. The use of adaptive walking modifications helps in maintaining life-space mobility and autonomy in participation in outdoor activities (Skantz et al., 2019). This is essential since higher life-space mobility is associated with better quality of life among older people (Rantakokko et al., 2013).

In the present study, perceiving nature- and infrastructure-related environmental outdoor mobility barriers was associated with a higher likelihood for both adaptive and maladaptive walking modifications in the age- and sex-adjusted models. However, the associations across the individual environmental outdoor mobility barriers were not identical and most were attenuated when health and physical capacity were added into the models. For instance, reporting snow and ice in the winter as a barrier increased the odds for using adaptive, but not maladaptive, walking modifications. Unlike those who have given up or reduced their frequency of walking longer distances, older people with adaptive walking modifications are likely to walk outdoors during wintertime, and thus perceive snow and ice as barriers that can be overcome (Eronen et al., 2014b).

In our prospective analyses, perceived environmental outdoor mobility facilitators did not predict the use of adaptive or maladaptive walking modifications. The sole exception was that reporting a walking trail or skiing track as a facilitator for outdoor mobility protected the individual from developing maladaptive walking modifications over time. Moreover, when health and physical capacity were included in the models, none of the perceived environmental outdoor mobility barriers increased the risk for using maladaptive walking modifications over time. These weak and unsystematic prospective associations indicate that perceptions of environmental characteristics do not necessarily precede the onset of walking modifications. However, this finding seems to be reasonable. Perceiving outdoor mobility facilitators decreases the risk for functional decline over time, while at the same time, perceiving facilitators encourages the use of adaptive rather than maladaptive walking modifications, thereby weakening longitudinal associations.

In the present study, adjusting the models for physical capacity and other health characteristics attenuated most of the associations between the environmental barriers to outdoor mobility and walking modifications. This finding underlines the importance of individual characteristics in person–environment fit models. This was also supported by our sensitivity analyses, which showed that the exclusion of participants who were unable to walk 2 km independently attenuated most of the associations between the environmental facilitators for outdoor mobility and walking modifications. Thus, in line with ecological model of aging (Nahemow & Lawton, 1973), the use of adaptive and maladaptive walking modifications seems to be the result of person–environment interaction. When older people with intermediate physical capacity start to perceive environmental barriers, they are able to overcome them by modifying their walking in an adaptive way and thus continue walking. However, as their physical capacity further declines, environmental press increases and compensation for functional loss via adaptive walking modifications is more difficult. In such a situation, because compensation requires at least some resources (Saajanaho et al., 2016), older people may turn to loss-based selection (Baltes & Baltes, 1990) and use maladaptive walking modifications. Previous studies have shown that multiple factors, such as age, family context, and functional capacity, are associated with the use of compensatory strategies (Gitlin et al., 2017; Hoenig et al., 2006; Lang et al., 2002). Our analyses complement these factors with that of the outdoor environment, which, depending on the individual’s level of physical or psychological functioning, seems to have specific impacts on the use of walking modifications.

The strengths of this study are the large population-based sample, with a 2-year follow-up, and the LISPE study design, which was optimized for the purpose of investigating the associations between environmental factors and outdoor mobility. However, the study has some limitations. First, perceptions of environmental facilitators for and barriers to outdoor mobility are individuals’ subjective feelings about their living environment and are expressed differently in different contexts. For example, our findings concern community-dwelling older adults mainly living in urban or suburban areas and hence might not be applicable to older adults living in rural areas. Second, participants relocating or experiencing changes in their living environment during the follow-up period might have had a minor effect on our longitudinal findings. It seems reasonable to expect that older people who relocate are likely to move from a more to a less challenging environment. If so, this might attenuate the longitudinal results. However, only 31 participants relocated during the follow-up and thus, any such effect is likely to be small. Similarly, it is possible that during the follow-up changes in the built environment, such as changes related to the availability of benches or to improvements or deterioration in sidewalks, or changes in the natural environment may have influenced the adoption of walking modifications. However, such changes in the study area were minor and not likely to have exerted a major impact on the longitudinal findings. Third, based on their SPPB scores, our participants were relatively well-functioning older people. This may have led to underestimation of the use of maladaptive walking modifications in the community-dwelling older population. However, the main purpose was to study the associations between features of the outdoor environment and walking modifications rather than the prevalence of walking modifications. Moreover, task limitations initially affect the most demanding tasks, such as walking longer distances (Weiss et al., 2007), and therefore using a measure of walking modifications in walking a distance of 2 km was appropriate in this group. Finally, our results may have been influenced by the fact that older people with severe mobility limitations rarely report environmental outdoor mobility barriers (Eronen et al., 2014a) owing to their lack of exposure to such barriers and hence unawareness of them.

## Conclusion

Whereas previous research findings have mainly concerned individual determinants of adaptive strategies, the present study, in line with the ecological model of aging, shows that the use of adaptive and maladaptive walking modifications seems to be the result of the person–environment interaction. Older people with adaptive walking modifications reported more environmental facilitators to outdoor mobility than people using maladaptive walking modifications. This indicates that perceived environmental facilitators, such as the availability of good quality walkways and good lighting, motivate individuals to continue walking in an adaptive way despite functional decline. The present finding of an association between perceived environmental barriers to outdoor mobility and the use of maladaptive walking modifications highlights the importance of a safe and walkable environment for increasing outdoor mobility among older people. It would, therefore, be prudent to reduce environmental barriers, especially for those with poorer physical capacity. For example, ensuring snow removal during wintertime (in localities with persistent snowy conditions) and providing resting places in streets and parks would benefit this group of people.

## Supplemental Material

Supplemental_tables – Supplemental Material for Associations between Perceived Outdoor Environment and Walking Modifications in Community-Dwelling Older People: A Two-Year Follow-Up Study Click here for additional data file. Supplemental Material, Supplemental_tables for Associations between Perceived Outdoor Environment and Walking Modifications in Community-Dwelling Older People: A Two-Year Follow-Up Study by Heidi Skantz, Taina Rantanen, Timo Rantalainen, Kirsi E. Keskinen, Lotta Palmberg, Erja Portegijs, Johanna Eronen and Merja Rantakokko in Journal of Aging and Health
