# Supplementary material for: Associations between Perceived Outdoor Environment and Walking Modifications in Community-Dwelling Older People: A Two-Year Follow-Up Study
Source: J Aging Health. 2020 Jul 28;32(10):1538–51. doi: 10.1177/0898264320944289 (PMC8688982; doi:10.1177/0898264320944289)
Supplement: Supplemental_tables – Supplemental Material for Associations between Perceived Outdoor Environment and Walking Modifications in Community-Dwelling Older People: A Two-Year Follow-Up Study [file Supplemental_tables.pdf]

Supplementary Table 1. Sensitivity Analyses. Cross-sectional Associations of Perceived Environmental Facilitators for Outdoor Mobility with Walking Modifications in Community-Dwelling Older People. Odds are Reported for those with No Modifications (n = 280) and Adaptive Modifications (n = 315) vs. Maladaptive Modifications (n = 123, reference).

	Model 1				Model 2			
	No walking modifications (n = 280)		Adaptive walking modifications (n = 315)		No walking modifications (n = 280)		Adaptive walking modifications (n = 315)	
**Facilitators**	OR (95 % CI)	Adjusted p-value	OR (95 % CI)	Adjusted p-value	OR (95 % CI)	Adjusted p-value	OR (95 % CI)	Adjusted p-value
**Sum of nature facilitators**								
1 vs. 0	0.9 (0.4–1.9)	0.906	0.6 (0.3–1.1)	0.166	0.8 (0.4–1.8)	0.761	0.5 (0.3–1.0)	0.194
≥ 2 vs. 0	1.9 (1.0–3.9)	0.141	0.9 (0.5–1.7)	0.841	1.8 (0.9–3.8)	0.289	0.9 (0.5–1.7)	0.821
**Sum of infrastructure facilitators**								
1 vs. 0	1.3 (0.7–2.5)	0.565	1.2 (0.6–2.1)	0.793	1.4 (0.7–2.8)	0.516	1.2 (0.7–2.3)	0.709
≥ 2 vs. 0	1.5 (0.9–2.5)	0.265	1.5 (0.9–2.4)	0.242	1.6 (0.9–2.9)	0.253	1.6 (0.9–2.6)	0.246
**Sum of safety facilitators**								
1 vs. 0	0.6 (0.3–1.4)	0.372	1.6 (0.8–3.4)	0.323	0.6 (0.3–1.4)	0.398	1.6 (0.8–3.4)	0.402
≥ 2 vs. 0	1.4 (0.8–2.7)	0.377	1.8 (0.9–3.3)	0.157	1.5 (0.8–3.0)	0.417	1.9 (1.0–3.6)	0.170
**Item-specific**								
Nature								
Park or other green area	1.2 (0.8–1.9)	0.606	1.1 (0.8–1.8)	0.674	1.2 (0.7–1.9)	0.709	1.1 (0.7–1.8)	0.733
Walking trail, skiing track	**2.8 (1.8–4.4)**	**<0.001**	1.3 (0.8–1.9)	0.377	**2.8 (1.7–4.5)**	**<0.001**	1.3 (0.9–2.0)	0.383
Nature, lakeside	1.2 (0.7–2.1)	0.589	0.9 (0.5–1.4)	0.658	1.2 (0.7–2.1)	0.709	0.9 (0.5–1.4)	0.709
Infrastructure								
Good lighting	1.4 (0.9–2.2)	0.273	1.3 (0.8–2.0)	0.404	1.4 (0.9–2.2)	0.377	1.3 (0.8–2.0)	0.417
Peaceful and good quality walkways	1.6 (1.0–2.5)	0.097	1.4 (0.9–2.1)	0.252	1.7 (1.0–2.7)	0.133	1.5 (1.0–2.3)	0.176
Even sidewalks	0.9 (0.5–1.4)	0.756	1.4 (0.9–2.1)	0.311	1.0 (0.6–1.7)	0.971	1.4 (0.9–2.3)	0.290
Resting places by the walking route	0.8 (0.5–1.4)	0.645	1.3 (0.8–2.2)	0.442	1.0 (0.6–1.9)	0.945	1.4 (0.8–2.3)	0.393
Walkways without steep hills	1.1 (0.5–2.1)	0.964	1.5 (0.8–2.8)	0.313	1.5 (0.7–3.1)	0.513	1.8 (0.9–3.5)	0.213
Services close	1.4 (0.9–2.2)	0.252	1.3 (0.9–2.0)	0.343	1.4 (0.9–2.3)	0.311	1.3 (0.9–2.0)	0.388
Safe crossings: Traffic lights, zebra crossing or traffic island between lanes	1.2 (0.7–2.0)	0.624	1.4 (0.9–2.3)	0.295	1.2 (0.7–2.1)	0.672	1.5 (0.9–2.4)	0.302
Safety								
Familiar environment	**1.8 (1.2–2.9)**	**0.030**	1.3 (0.9–2.1)	0.295	**2.0 (1.2–3.2)**	**0.028**	1.4 (0.9–2.2)	0.265
Appealing scenery	1.3 (0.8–2.1)	0.370	1.1 (0.7–1.7)	0.887	1.4 (0.9–2.4)	0.352	1.1 (0.7–1.7)	0.817
Own yard	1.0 (0.7–1.6)	0.977	1.1 (0.8–1.7)	0.691	0.9 (0.6–1.4)	0.768	1.2 (0.8–1.9)	0.584
Other people outdoors motivate	1.3 (0.8–2.3)	0.442	1.2 (0.7–2.0)	0.645	1.7 (0.9–3.0)	0.239	1.4 (0.8–2.3)	0.458
No car traffic	1.1 (0.6–2.1)	0.841	1.0 (0.6–1.9)	0.972	1.2 (0.6–2.4)	0.731	1.1 (0.6–2.0)	0.878
No cyclists on walkways	0.8 (0.3–1.9)	0.691	0.8 (0.3–1.9)	0.691	0.7 (0.3–2.0)	0.709	0.8 (0.3–1.9)	0.709

Note: Multinomial logistic regression analyses. Reference category: maladaptive walking modifications, n = 123. Model 1: Adjusted for age and sex. Model 2: Adjusted for age, sex, years of education, depressive symptoms, chronic conditions, and lower extremity function. OR = Odds Ratio; CI = Confidence Interval. False discovery rates (adjusted p-values) were calculated to correct for multiple testing.

Supplementary Table 2. Sensitivity Analyses. Cross-sectional Associations of Perceived Environmental Barriers to Outdoor Mobility with Walking Modifications among Community-Dwelling Older People. Odds are Reported for those with Adaptive Modifications (n = 315) and Maladaptive Modifications (n = 123) vs. those with No Modifications (n = 280, reference).

	Model 1				Model 2			
	Adaptive walking modifications (n = 315)		Maladaptive walking modifications (n = 123)		Adaptive walking modifications (n = 315)		Maladaptive walking modifications (n = 123)	
**Barriers**	OR (95 % CI)	Adjusted p-value	OR (95 % CI)	Adjusted p-value	OR (95 % CI)	Adjusted p-value	OR (95 % CI)	Adjusted p-value
**Sum of nature barriers**								
1 vs. 0	**2.4 (1.6–3.4)**	**<0.001**	1.8 (1.1–2.9)	0.045	**1.8 (1.2–2.7)**	**0.020**	1.1 (0.7–1.9)	0.768
2 vs. 0	**4.9 (2.9–8.5)**	**<0.001**	2.2 (1.1–4.5)	0.087	**3.6 (2.0–6.5)**	**<0.001**	1.4 (0.6–2.9)	0.620
**Sum of infrastructure barriers**								
1 vs. 0	**1.6 (1.1–2.5)**	0.087	1.9 (1.1–3.2)	0.074	1.4 (0.9– 2.1)	0.383	1.5 (0.8 –2.7)	0.377
≥ 2 vs. 0	**4.0 (2.4–6.7)**	**<0.001**	**3.7 (2.0–7.0)**	**<0.001**	**2.6 (1.5–4.6)**	**0.006**	**2.2 (1.1–4.3)**	0.094
**Sum of safety barriers**								
1 vs. 0	1.5 (0.9–2.3)	0.187	1.0 (0.5–1.8)	0.968	1.2 (0.8–2.0)	0.609	0.8 (0.4–1.5)	0.682
≥ 2 vs. 0	**2.3 (1.3–4.0)**	**0.012**	1.2 (0.6–2.6)	0.756	1.5 (0.8–2.6)	0.401	0.7 (0.3–1.6)	0.542
**Item-specific**								
Nature								
Hills in the nearby environment	**2.6 (1.6–4.0)**	**<0.001**	1.9 (1.1–3.3)	0.090	**2.1 (1.3–3.3)**	**0.015**	1.5 (0.8–2.7)	0.402
Snow and ice in winter	**2.9 (2.0–4.0)**	**<0.001**	1.6 (1.0–2.5)	0.090	**2.2 (1.5–3.2)**	**<0.001**	1.1 (0.7–1.7)	0.894
Infrastructure								
Poor street condition	1.5 (1.0–2.3)	0.140	0.8 (0.4–1.5)	0.645	1.2 (0.7–1.9)	0.709	0.5 (0.3–1.1)	0.210
High curbs	**3.6 (1.4–9.1)**	**0.023**	2.3 (0.7–7.3)	0.268	2.2 (0.8–6.1)	0.298	1.1 (0.3–3.9)	0.941
Lack of pedestrian zones	2.2 (0.8–6.5)	0.268	2.4 (0.7–8.8)	0.295	2.9 (0.9–9.8)	0.226	3.3 (0.8–13.8)	0.254
Long distances to services	2.1 (1.0–4.2)	0.119	**4.7 (2.2–9.8)**	**<0.001**	1.8 (0.8–3.9)	0.298	**4.0 (1.7–9.1)**	**0.006**
Lack of resting places, summer	**3.4 (1.9–6.0)**	**<0.001**	**4.0 (2.0–7.9)**	**<0.001**	2.1 (1.1–4.0)	0.072	2.4 (1.1–4.8)	0.080
Lack of resting places, winter	**3.5 (2.0–5.9)**	**<0.001**	**3.4 (1.8–6.3)**	**<0.001**	**2.4 (1.4–4.3)**	**0.015**	2.2 (1.1–4.3)	0.102
Poor lighting	2.7 (1.0–7.2)	0.119	1.3 (0.3–5.4)	0.841	2.2 (0.8–6.2)	0.320	1.0 (0.2–4.3)	0.962
Safety								
Noisy traffic	3.2 (1.2–8.9)	0.078	2.8 (0.8–9.7)	0.197	2.0 (0.7–6.2)	0.389	2.0 (0.5–7.4)	0.498
Busy traffic	2.4 (1.2–4.6)	0.045	2.2 (0.9–5.0)	0.152	1.7 (0.8–3.6)	0.302	1.6 (0.6–3.8)	0.516
Dangerous crossroads	2.0 (1.1–3.6)	0.076	1.3 (0.6–2.9)	0.712	1.6 (0.8–2.9)	0.352	1.0 (0.4–2.3)	0.945
Vehicles on walkways	1.1 (0.3–4.2)	0.972	1.0 (0.2–6.0)	0.995	0.8 (0.2–3.6)	0.886	0.7 (0.1–4.6)	0.821
Cyclists in the walkways	1.5 (0.9–2.3)	0.142	0.7 (0.4–1.4)	0.502	1.2 (0.7–1.8)	0.715	0.5 (0.3–1.0)	0.194
Insecurity due to other pedestrians	1.7 (0.8–3.4)	0.295	0.9 (0.3–2.7)	0.968	1.0 (0.4–2.2)	0.971	0.5 (0.2–1.6)	0.417

Note: Multinomial logistic regression analyses. Reference category: no walking modifications, n = 280. Model 1: Adjusted for age and sex. Model 2: Adjusted for age, sex, years of education, depressive symptoms, chronic conditions, and lower extremity function. OR = Odds Ratio; CI = Confidence Interval. False discovery rates (adjusted p-values) were calculated to correct for multiple testing.

Supplementary Table 3. Sensitivity Analyses. Perceived Environmental Facilitators for Outdoor Mobility as Predictors of Use of Maladaptive Walking Modifications over the 2-Year Follow-Up in Community-Dwelling Older People (N = 605).

	Maladaptive walking modifications			
	Model 1		Model 2	
**Facilitators**	OR (95 % CI)	Adjusted p-value	OR (95 % CI)	Adjusted p-value
**Sum of nature facilitators**				
1 vs. 0	0.7 (0.4–1.1)	0.268	0.7 (0.4–1.7)	0.277
≥ 2 vs. 0	0.9 (0.5–1.7)	0.931	1.0 (0.5–1.7)	0.910
**Sum of infrastructure facilitators**				
1 vs. 0	0.9 (0.6–1.5)	0.892	0.9 (0.5–1.6)	0.664
≥ 2 vs. 0	0.9 (0.5–1.5)	0.841	0.9 (0.5–1.6)	0.843
**Sum of safety facilitators**				
1 vs. 0	1.0 (0.6–1.8)	0.980	0.9 (0.5–1.7)	0.857
≥ 2 vs. 0	1.9 (0.9–3.7)	0.154	1.7 (0.8–3.3)	0.329
**Item-specific**				
Nature				
Park or other green area	0.8 (0.6–1.2)	0.242	0.7 (0.5–1.1)	0.258
Walking trail, skiing track	**0.5 (0.4–0.7)**	**<0.001**	**0.5 (0.3–0.7)**	**<0.001**
Nature, lakeside	1.2 (0.8–1.9)	0.471	1.3 (0.8–1.9)	0.481
Infrastructure				
Good lighting	0.9 (0.7–1.3)	0.841	0.9 (0.6–1.3)	0.761
Peaceful and good quality walkways	0.9 (0.6–1.2)	0.645	0.9 (0.6–1.2)	0.584
Even sidewalks	1.3 (0.9–1.8)	0.353	1.2 (0.8–1.7)	0.584
Resting places by the walking route	1.5 (1.0–2.3)	0.123	1.4 (0.9–2.1)	0.330
Walkways without steep hills	1.2 (0.7–1.9)	0.661	1.1 (0.7–1.9)	0.777
Services close	1.2 (0.8–1.6)	0.598	1.2 (0.8–1.7)	0.540
Safe crossings: Traffic lights, zebra crossing or traffic island between lanes	0.9 (0.6–1.3)	0.658	0.8 (0.6–1.3)	0.584
Safety				
Familiar environment	1.0 (0.7–1.5)	0.972	1.0 (0.7–1.5)	0.945
Appealing scenery	0.8 (0.5–1.1)	0.268	0.8 (0.5–1.1)	0.321
Own yard	1.1 (0.8–1.6)	0.701	1.1 (0.8–1.6)	0.709
Other people outdoors motivate	0.9 (0.6–1.4)	0.897	0.9 (0.6–1.3)	0.642
No car traffic	0.9 (0.6–1.5)	0.892	0.9 (0.5–1.4)	0.731
No cyclists on walkways	1.1 (0.5–2.5)	0.917	1.0 (0.4–2.4)	0.971

Note: Development of maladaptive walking modifications was analyzed by using binary logistic regression models. Reference category: no and adaptive walking modifications. Model 1: Adjusted for age and sex. Model 2: Adjusted for age, sex, years of education, depressive symptoms, chronic conditions, and lower extremity function. OR = Odds Ratio; CI = Confidence Interval. False discovery rates (adjusted p-values) were calculated to correct for multiple testing.

Supplementary Table 4. Sensitivity Analyses. Perceived Environmental Barriers to Outdoor Mobility as Predictors of Use of Maladaptive Walking Modifications over 2-Year Follow-Up in Community-Dwelling Older People (N = 605).

	Maladaptive walking modifications			
	Model 1		Model 2	
**Barriers**	OR (95 % CI)	Adjusted p-value	OR (95 % CI)	Adjusted p-value
**Sum of nature barriers**				
1 vs. 0	**2.5 (1.5–4.1)**	**<0.001**	2.0 (1.2–3.4)	0.046
2 vs. 0	**1.7 (1.1–2.5)**	**0.030**	1.4 (0.9–2.1)	0.288
**Sum of infrastructure barriers**				
1 vs. 0	1.6 (1.0–2.5)	0.119	1.3 (0.8–2.1)	0.536
≥ 2 vs. 0	1.2 (0.7–1.8)	0.661	1.1 (0.7–1.7)	0.900
**Sum of safety barriers**				
1 vs. 0	1.1 (0.7–2.0)	0.750	0.9 (0.5–1.6)	0.836
≥ 2 vs. 0	1.4 (0.9–2.2)	0.229	1.3 (0.8–2.1)	0.418
**Item-specific**				
Nature				
Hills in the nearby environment	**1.7 (1.1–2.7)**	**0.040**	1.6 (1.0–2.4)	0.170
Snow and ice in winter	**1.8 (1.3–2.6)**	**0.004**	1.5 (1.1–2.2)	0.102
Infrastructure				
Poor street condition	1.5 (0.9–2.3)	0.147	1.3 (0.8–2.0)	0.436
High curbs	1.7 (0.8–3.5)	0.273	1.2 (0.6–2.6)	0.777
Lack of pedestrian zones	1.0 (0.3–3.2)	0.978	1.3 (0.4–4.2)	0.768
Long distances to services	1.0 (0.5–2.0)	0.995	1.0 (0.5–1.9)	0.945
Lack of resting places, summer	1.4 (0.8–2.3)	0.372	1.1 (0.6–1.8)	0.878
Lack of resting places, winter	1.6 (1.0–2.5)	0.141	1.3 (0.8–2.1)	0.533
Poor lighting	1.1 (0.4–2.6)	0.969	0.8 (0.3–2.0)	0.761
Safety				
Noisy traffic	1.0 (0.4–2.5)	0.977	0.7 (0.3–1.9)	0.664
Busy traffic	1.9 (1.0–3.6)	0.108	1.6 (0.8–3.1)	0.318
Dangerous crossroads	1.2 (0.7–2.2)	0.645	1.1 (0.6–1.9)	0.886
Vehicles on walkways	0.9 (0.2–3.6)	0.917	0.8 (0.2–3.4)	0.851
Cyclists in the walkways	1.3 (0.8–1.9)	0.440	1.2 (0.7–1.8)	0.709
Insecurity due to other pedestrians	1.0 (0.5–2.1)	0.977	0.8 (0.4–1.7)	0.761

Note: Development of maladaptive walking modifications was analyzed by using binary logistic regression models. Reference category: no and adaptive walking modifications. Model 1: Adjusted for age and sex. Model 2: Adjusted for age, sex, years of education, depressive symptoms, chronic conditions and lower extremity function. OR = Odds Ratio; CI = Confidence Interval. False discovery rates (adjusted p-values) were calculated to correct for multiple testing.
